# Zygmunt Bauman. Individual and society in the liquid modernity

**DOI:** 10.1186/2193-1801-2-191

**Published:** 2013-04-29

**Authors:** Emma Palese

## Abstract

Starting from the postmodern, the philosophical and sociological speculation by Zygmunt Bauman, opens - through the analysis of the phenomenon of globalization – to the meta-level of life, and then circumscribes the most recent thinking on political life, until reaching the liquid modernity: overcoming postmodernity itself. As a result individual, society, ethics, power, religion become those words impregnated with a liquidity capable of condensing in itself the most significant aspects of the present reality: a dimension in which the lasting gives way to the transient, the need to the desire, and the necessity to the utility.

Zygmunt Bauman is one of the greatest interpreters of our present time, a time which turns into a shapeless mass tending to a constant and relentless change. This is not the modern era, nor the postmodern one, if anything, this period can be well identified as liquid modernity: a concept, able to focus on the transformations that affect human life concerning the general policy determinations of life. Moreover, Bauman’s liquid modernity is a term that can overcome the concept of postmodernism, because basically leaning towards the contemporary world: the reality in which life considers highly what is transitory rather than permanent, the immediate rather than long term; and regards utility as prior to any other value. Consequently it is fundamental to understand in advance and profoundly the concept of liquidity around which Bauman interweaves his most recent philosophical and sociological reflection. Solidity and liquidity are the distinctive features of two eras: modernity and postmodernity, which becomes liquid modernity as it relates to contemporary existence. It is an existence where the need gives way to the desire that dismays men in the constant changes and transformations that affect their lives, and that turn identity from fact into a task: each of us runs into the self-building, which replaces the project itself. Indeed, in our contemporary age the relationship between the individual and society is changing because the concepts of identity, individual and individuality are becoming meaningless. The world demands to the individual a constant and increasingly controversial search for identity and tracking of parameters for standardizing in order to obtain the “role” of individuals, because, today, the identity is a task. Being individuals in the liquid society does not simply mean being good consumers, but also being competitive goods in the global market. Such condition does not only require the purchase of “fashion items”, but, also, the purchase of a “fashionable body” assisting to the complete passage from self-manipulation of our own physicality, to the real direct and independent choice of the body we want for our children. Structured on this pattern, Bauman’s futuristic view asserts that “being suitable for the global” will not be satisfied for long with plastic surgery and remodeling on the basis of *topoi* which are continuously generated by the policies of the global market. It is important not only to buy what makes us “suitable” for the contemporary, but above all to change ourselves, the closest part to our possibility of manipulation and intervention: the body. It becomes a free space on which to shape the visible Self, since if we are not able to dress up our own physical, it means that we are lacking in something. The autonomous management of our corporeality, personal responsibility, which bears the “responsibility of being individuals”, derives from the concept of having and not of being. To have means to possess because some form of control is capable of generating security in a world lacking in its solid points of reference. For this reason the conditions of having also falls on the body of contemporary man, who finds in it a form of certainty: to manipulate and control his physical means acting on what you are sure to possess. Incorporation and possession are part of having, that - in our times -is accomplished through consumerism as «The act of consumption is a form of having, perhaps the most important for today’s opulent industrial society. Consumption has ambivalent features: it relieves the anxiety, because what one has, could not be taken back, but it also requires that consumers increasingly consume, since the previous consumption soon loses its rewarding peculiarity».^a^ And this vicious spiral, which runs between the possession and consumption, is the most evident effect of what Bauman calls liquid modernity, which -unlike postmodernism - has a continuous relationship with the modernization process, which has its origins in modern times -but it prolongs and intensifies until it reaches the liquidity of our time, characterized by rampant consumerism.

And in the convergence between identity and consumption lies one of the main features of our age, because «contemporary society relates to its members primarily as consumers, and only secondarily, and in part, involves them also as producers. To meet the standards of normality and to be recognized as a mature and respectable members of society, we must respond quickly and efficiently to the temptations of the consumer goods market. It should be offered regularly a contribution to the demand fit to absorb the supply and, in the stages of reflection or stagnation of the economy, we must participate to the recovery led by consumers. The poor and the idlers, those who have neither a decent income, or credit cards, nor the prospect of better days, are not up to these requirements. Consequently the rule broken by the poor today, the violation of the rule distinguishes them and tags them as abnormal, is the standard of competence or fitness as consumers, not that of employment. The poor of today (ie those that constitute a problem for others) are first and foremost consumers rather than unemployed people. They are defined primarily by the fact of being bad consumers: indeed, the most basic of social obligations, which they do not comply, is the duty to be active and effective purchasers of goods and services offered by the market ».^b^ This means that if in a modern times consumption assumes the function of a secondary activity compared to production, in the contemporary world the ability of a person to consume determines his own social integration in a society no longer limited to the local context or the sheer size of the daily existence, but, in a macro society demanding accurate and specific entry requirements. And the access falls directly on the responsibility of the individual, who, in order to build his own individuality, prefers to invest its available economic resources for the purchase of those means suitable to classify, to modernize and introduce in the list of who matters. Moreover, in a period of severe economic crisis like the one we are currently experiencing, other statistics show that the primary consumption, referring to the basic necessities, is put aside to buy advanced technology products, clothing and cosmetics. These products aim at updating the body in accordance with the minimal standards required to be “in”, ie, to acquire a social status that does not differentiate, if anything, incorporates all those who appear to be able to modernize themselves, regardless of social productive capacity and the role everyone can play. Today consumption seems to be a homologating activity, and according to Bauman it is a way to measure how much a person - in liquid society - is able to be individual. On that concept, Bauman builds his thought about the individual and society, which runs on two main lines. The first is embodied in the idea that in the liquid world the conquest of identity goes hand in hand with the adherence to rules of a consumers society directed by the policies of the global market: being individuals is equivalent to being consumers. The second line, however, going further that consideration, expands to incorporate the individual in the products. The relationship between the individual and the self, as well as between the individual and the others, acquires through Z. Bauman’s thought - a new meaning, which is based on a real anthropological metamorphosis. Being consumers and being consumed become, in fact, most general determinations of an individual who is affected the most problematic effects of the process of de-socialization, started by globalization, now arrived at one of its most acute and extreme phases. Social aggregation and organization are deprived of their traditional tasks: they stop being identity dimensions of the subject capable of providing a set of standards and benchmarks. The individual becomes an isolated monad always looking for new forms of socialization, which instead of providing safety and welfare, increase the gap between man and the Self and between man and the other. It is a social system that - despite being in possession of increasingly innovative means to communicate and interact with their fellows - generates discomfort and loneliness, mainly because the son of a networked individualism that «it is a social model, not a collection of isolated individuals», just because «the most important role of internet in the structuring of social relations is the contribution to the new model of sociability based on individualism. More and more people are organized in social networks, which communicate via computer. Thus, it is not the internet to create a model of networked individualism, but the development of the Internet to provide adequate material support for the spread of individualism in the web as the dominant form of online socializing».^c^ And this new form arises as a matrix of the identity update required by the global world in order to “be included”, as the need for inclusion is nothing more than legacy of the abandonment of the authentic sense of belonging. The belonging, in fact, is characterized as a natural human feeling, that, being suppressed nowadays - is manifest in surrogate forms of virtual social aggregation that are the attempt to satisfy the natural human sociability. The consumer society, in fact, does not aggregate, at any rate, disaggregate turning groups into isolated monads, with weak and fragmented bonds, where the individual is crystallized between the search for Self and the disarray in the not - Self. The idea of society survives in terms of common trends to follow, where the groups are directed almost anonymously in the pursuit of that “happiness”, whose traces are designed by external actors. According to Bauman it is a review and revision of the “mechanical solidarity” in Durkheim,^d^ whose characteristics distinguish it from the “organic” one. The singularity and uniqueness of the individual is replaced by the flow of the needs of a group, which - in our contemporary world - seems to assume the appearance of a swarm. Just in the distinction between swarm and group Bauman identifies the radical changes that affect the individual and society in the liquid-consumerist reality, where «the swarm tends to replace the group and its leaders, its hierarchy and its “pecking order”. A swarm can do without all the ceremonial and tricks without which it would neither form nor survive. They come together, scatter and gather again, from an occasion to another, every time inevitably for a different reason, and are attracted by changeable aims. The seductive power of mobile objectives is a rule sufficient to coordinate movements, and this is enough to render superfluous any other command or imposition from above. In reality, the swarms do not even have a high and low: only the momentary direction of flight to place the units of the swarm (working self-propelled) in position of leader or followers, usually only for the duration of a given flight , or even a part of it». Then, even the traditional hierarchies that generate order, dissolve and become strong inviolable nucleus in which the individual can find himself, directing and limiting his own desires. This means that every opportunity to address the human being collapses. Consequently the individual is considered as a momentary unity of the passing swarm and driven by the fleeting current. That is a dimension impregnated with an illusory security of a free and optimal choice since it is the choice of a large number of people. The choice is what aggregates in a liquid world, as these spaces are to be rethought and redesigned according to certain canons able to shape the communities in which the individual - consumer can find and fulfill their sense of belonging. The malls seem to be hives of swarms of Bauman, as offering the ideally imagined community: a place where the purpose of purchasing aggregates. Thus, «the shopping /consumption places offer what no “real reality” outside can give: an almost perfect balance between liberty and security. Within their temples buyers / consumers may also find what they were searching outside, uselessly as inexhaustibly: the comfortable feeling of belonging, the reassuring impression of being part of a community».^e^ Taking part is one of the ways in which consumption becomes a primary activity of contemporary man, and – especially - the principle of inclusion and exclusion of the subject. Moreover, in this sense, Bauman tracks in the anthropophagic strategy theorized by Levi-Strauss,^f^ the practice of elimination of differences between individuals, which is reproduced in the supermarket: the privileged places of consumption in which is performed the aggregating power of purchase. Anthropophagic places contrast, in fact, to the “emic” ones, consisting in «vomiting and spitting the others out, considering them as being incurably strangers and aliens, in prohibiting physical contact, dialogue, social relationships and any kind of *commercium*, commensality or *connubium*. The extreme variants of this emic strategy are, today as always, imprisonment, deportation and physical suppression. Two updated forms, refined (modernized) are the spatial separation, urban ghettos, the selective access to spaces. The second strategy consists of a so-called “disalienation” of stranger substances: “in swallowing”, “eating” the bodies and the extraneous spirits to make them, through metabolism, identical and no longer distinguishable from the body that swallows them».^g^ Therefore, the consumption becomes a surrogate way for social gathering, which, however, replaces the sense of belonging with the need for inclusion. This process inevitably excludes those who are not in possession of the means fit to perform this activity, which, indeed, remains essentially solitary. Here in this game of appearances and reproductions the group gives way to the swarm, which, in the collective whirl, loses that authentic sense of belonging that makes each man a member of society, in which – mentioning Durkheim - it performs the natural duality of the subject: animal with socialized personality, union of instinct and reason, of self and world.

## Endnotes

^a^E. Fromm, *To have or to be?*, Harper e Row, New York, 1976, p. 40

^b^Z. Bauman, *Consuming life*, Polity Press, Cambridge, 2007, p. 157

^c^M. Castells*, Internet galaxy,* Oxford University press, 2001, p. 129; Idem*, The Rise of the Network Society*, Blackwell Publishers, Oxford, 1996 ; E. Susca*, Nuovi media, comunicazione, società,* Aras, Fano, 2007

^d^Durkheim thinks that social facts exist before the same individual because «the collective life, such as mental life is made of representations, so it is likely that individual representations and social representations are in any way comparable. We will try to show that in fact one and the other have the same relationship with the respective substrate. But this approach, far from justifying the view that sociology reduces to a simple corollary of individual psychology, will, on the contrary, stressed the relative independence of these two worlds of these two sciences. […] The representation is not a simple aspect of the state in which the item is nervous when cu occurs because when this was still not over and that the reports of the performances are of a different nature than that of underlying neural elements. […] The company's set of individuals associated with the substrate. The system which, together they form and vary depending on their number, their arrangement on the surface of a territory, the nature and number of lines of communication, forms the basis cu which elevates the social life». É. Durkheim, *Individual and Collective Representation (1898)*, in *Sociology and Philosophy*, Free Press, New York, 1974

^e^Z. Bauman, *Liquid modernity*, Polity Press, Cambridge, 2000, pp. 109-110

^f^Bauman considers Claude Levi Strauss the greatest anthropologist of our time, for wich «the fundamental principle is that the concept of social structure does not refer to empirical reality, but the models built upon it. It is therefore clear difference between the two concepts are so close that they were often confused, namely those of social structure and social relations. Social relationships are the raw material used for the construction of models that make manifest the social structure. In no case, therefore, it can be identified as the set of social relationships observable in a given society. The investigations of the structure do not claim a sphere of its own, between the facts of companies; rather constitute a method capable of being applied to various problems ethnological, and resemble forms of structural analysis in use in different fields. The important thing is to know that what constitutes those models that are the peculiar object of structural analysis. The problem is not ethnological, but epistemological, since the definitions that follow are independent of the raw material of our research. We think, that, to merit the name of the structure, models should only meet four conditions. First, a structure has the character of a system. It consists of elements such that any modification of one of them involves a modification of all the others. Secondly, each model belongs to a group of transformations each of which corresponds to a model of the same family, so that the set of such transformations constitute a group of models. Thirdly, the properties indicated above allow to predict how the model will react, in case of modification of one of its elements. Finally, the model must be constructed such that its functioning can explain all the observed facts ». C. Lévi - Strauss, *Anthropologie structurale*, Librairie Plon, Paris, 1964, pp. 311-12

^g^Z. Bauman, *Liquid Modernity*, op. cit., p. 112
